# Fabrication of Customizable Intraplaque Hemorrhage Phantoms for Magnetic Resonance Imaging

**DOI:** 10.1007/s11307-022-01722-4

**Published:** 2022-04-29

**Authors:** Matteo A. Bomben, Alan R. Moody, James M. Drake, Naomi Matsuura

**Affiliations:** 1 Department of Mechanical and Industrial Engineering, University of Toronto, Toronto, ON Canada; 2 The Wilfred and Joyce Posluns Centre for Image Guided Innovation and Therapeutic Intervention, The Hospital for Sick Children, Toronto, ON Canada; 3 Department of Medical Imaging, University of Toronto, Toronto, ON Canada; 4 Sunnybrook Hospital, Toronto, ON Canada; 5 Institute of Biomedical Engineering, University of Toronto, 184 College Street, Room 140, Toronto, ON M5S 3E4 Canada; 6 Department of Materials Science and Engineering, University of Toronto, Toronto, ON Canada

**Keywords:** Intraplaque hemorrhage, MRI phantoms, Methemoglobin, Carotid plaque, MR imaging, Magnetic resonance angiography

## Abstract

**Purpose:**

Magnetic resonance (MR) imaging detection of methemoglobin, a molecular marker of intraplaque hemorrhage (IPH), in atherosclerotic plaque is a promising method of assessing stroke risk. However, the multicenter imaging studies required to further validate this technique necessitate the development of IPH phantoms to standardize images acquired across different scanners. This study developed a set of phantoms that modeled methemoglobin-laden IPH for use in MR image standardization.

**Procedures:**

A time-stable material mimicking the MR properties of methemoglobin in IPH was created by doping agarose hydrogel with gadolinium and sodium alginate. This material was used to create a phantom that consisted of 9 cylindrical IPH sites (with sizes from 1 to 8 mm). Anatomical replicas of IPH-positive atherosclerosis were also created using 3D printed molds. These plaque replicas also modeled other common plaque components including a lipid core and atheroma cap. T1 mapping and a magnetization-prepared rapid acquisition gradient echo (MPRAGE) carotid imaging protocol were used to assess phantom realism and long-term stability.

**Results:**

Cylindrical phantom IPH sites possessed a T1 time of 335 ± 51 ms and exhibited little change in size or MPRAGE signal intensity over 31 days; the mean (SD) magnitude of changes in size and signal were 6.4 % (2.7 %) and 7.3 % (6.7 %), respectively. IPH sites incorporated into complex anatomical plaque phantoms exhibited contrast comparable to clinical images.

**Conclusions:**

The cylindrical IPH phantom accurately modeled the short T1 time characteristic of methemoglobin-laden IPH, with the IPH sites exhibiting little variation in imaging properties over 31 days. Furthermore, MPRAGE images of the anatomical atherosclerosis replicas closely matched those of clinical plaques. In combination, these phantoms will allow for IPH imaging protocol standardization and thus facilitate future multicenter IPH imaging.

**Supplementary Information:**

The online version contains supplementary material available at 10.1007/s11307-022-01722-4.

## Introduction

Intraplaque hemorrhage (IPH) is a commonly observed feature of atherosclerotic plaque and an indicator of plaque instability [1–4]. Carotid magnetic resonance imaging (MRI) has been shown to detect IPH-positive plaques and thereby identify patients with an elevated stroke risk [5, 6]. Plaque-based deposits of methemoglobin, a molecular marker of IPH [7] and a hemoglobin derivative, are paramagnetic and thus cause substantial T1-shortening [8] permitting easy detection using heavily T1-weighted, magnetization-prepared rapid gradient echo (MPRAGE) imaging [9]. Furthermore, by nulling the signal from blood and employing the water excitation technique to remove the surrounding subcutaneous fat, maximum contrast between background tissue and the methemoglobin-induced signal has been achieved on MPRAGE images [7, 10]. This technique has detected IPH with over 95 % specificity and high sensitivity when compared to a histological gold standard [9].

Despite these initial successes, MPRAGE-based IPH detection has yet to gain widespread use as a stroke-risk assessment tool. Previous studies have typically been performed using a specific model of scanner within a single institution [11, 12]. Large, multicenter studies using a range of MRI platforms are a key step towards greater clinical adoption. The potential variability of magnetic resonance (MR) images acquired using different MRI platforms will impact studies seeking to quantify IPH characteristics such as volume or signal intensity. Inter-scanner variability in multicenter studies can be overcome by using imaging phantoms to calibrate scanners and ensure standardized images [13–15]. However, currently available atherosclerosis MRI phantoms fail to model IPH as they do not exhibit the hyperintense regions associated with methemoglobin buildup [16–18]. To allow for the further development of MR imaging as a clinical tool in stroke assessment, there exists a clear need for MRI phantoms that accurately model the appearance of IPH in atherosclerosis, in terms of both MR relaxation properties and shape.

A primary requirement of such phantoms is that the size and MR relaxation properties of the IPH being modeled remain constant and spatially homogenous over the time required for scanner calibration. In the context of multicenter studies, this may include phantom transport between institutions. Although doping hydrogels (commonly used as tissue mimics in MRI [19, 20]) with gadolinium can mimic the T1-shortening effects of methemoglobin, such a strategy may not be well-suited for use in IPH phantoms as Gd^3+^ ions can diffuse/migrate through these gels unless they are bound to the gels’ polymer matrix or otherwise immobilized. Such diffusion/migration would cause phantom T1 relaxation times to change over time as Gd^3+^ ions migrate from the mock IPH site into other regions of the phantom. While some phantoms are able to prevent the above-described migration by encasing each region in an impermeable barrier (*e.g.,* plastic or glass) [21], these barriers are visible on MR images and thus cause phantoms that seek to re-create anatomical/biological structures to appear less realistic. Furthermore, encasing all components of a phantom in a plastic or glass barrier also decreases a phantom’s manufacturability, especially for those with small or complex features. For these reasons, such a strategy could not be used in the design of IPH-positive atherosclerosis phantoms which, in order to appear realistic, should have adjacent regions (*e.g.,* IPH and vessel wall) in direct contact with each other.

The objective of this research was to develop a novel set of realistic IPH phantoms that could be used to calibrate MRI scanners and would thus facilitate multi-site IPH imaging studies. First, a methemoglobin-mimicking material whose T1 relaxation properties remained near-constant over time was synthesized. To accomplish this, sodium alginate, an anionic polysaccharide, was incorporated into gadolinium-doped agarose hydrogels so as to immobilize the gadolinium cations within the cross-links that can form between chains of the alginate polymer in the presence of polyvalent metal cations [22]. This material was then used to create two distinct phantoms that could be used in scanner calibration: one that mimics cylindrical IPH sites of varying size (*i.e.,* an IPH size standard) so that IPH area/volume measurements can be more easily calibrated, and a second that models an anatomical atherosclerotic plaque possessing IPH. To demonstrate the clinical utility of the phantoms, their long-term stability and the realism of their appearance were assessed via T1 mapping and MPRAGE IPH imaging.

## Materials and Methods

### Methemoglobin-Mimicking Material

A hydrogel composed of 1.5 wt % agarose and 0.15 wt % sodium alginate was doped with 0.05 wt % sodium azide (to prevent fungal growth) and gadolinium(III) chloride hexahydrate (as a T1 shortening agent). Gadolinium was added to ensure the material exhibited the short T1 time characteristic of the methemoglobin deposits present in IPH. Formulations containing 0.015 wt %, 0.005 wt %, and 0.0027 wt % gadolinium(III) chloride hexahydrate were tested in order to ensure that the long-term stability of the material’s T1 relaxation properties was not dependent on gadolinium concentration.

Sodium alginate–containing hydrogel formulations were assessed to evaluate if the material is indeed effective at preventing Gd^3+^ migration and if the T1 properties remained constant over time, even when in direct contact with another hydrogel. Each methemoglobin-mimicking hydrogel was cast into a cylindrical shape (possessing a diameter of 20.7 mm) and encased in a block of 2% agar gel. This produced a high surface area interface between the methemoglobin-mimicking material and the surrounding agar gel. To demonstrate the role sodium alginate plays in preventing Gd^3+^ migration, the alginate-containing methemoglobin hydrogels were tested in comparison to alginate-free gels. After fabrication, this experimental setup was stored in deionized water at room temperature. T1 relaxation maps and MPRAGE images (see details below) of the experimental setup were acquired every 7 days for a 42-day period. The mean T1 relaxation time of each methemoglobin-mimicking hydrogel was calculated, and MPRAGE images were used to plot signal contrast (*versus* the agar background) as a function of radial position in the cylindrical hydrogel.

### IPH Size Standard Phantom

A phantom consisting of nine cylindrical mock-IPH sites (with diameters of 1, 1.5, 2, 2.5, 3, 4, 5, 6, and 8 mm) was embedded in an agar hydrogel. To create the phantom, nine stainless steel rods, with the diameters indicated above, were suspended across a custom-made box and a 2 wt % agar hydrogel was cast around them. The rods were removed, producing a set of hollowed tubular volumes, and the methemoglobin-mimicking hydrogel (with a gadolinium(III) chloride hexahydrate concentration of 0.01 wt %) was injected into these hollowed volumes to produce the IPH sites. The completed phantom was imaged following fabrication and after a 31-day storage period (in deionized water at room temperature) using the MPRAGE sequence and a T1 mapping protocol (Siemens VIBE, see below). The resulting images were used to determine the initial T1 time of the mock IPH sites, the mean cross-sectional area of each site (before and after storage), and the percent change in IPH signal intensity over the storage period.

### Anatomical Atherosclerosis Phantom

A mock vessel with a lumen diameter of 8 mm and wall thickness of 2 mm was fabricated. The vessel was cast from a 2 wt % agar solution using a 3D printed core–shell mold (Objet500 Connex3, Stratasys, Eden Prairie, MN). Paraffin wax was cast around the vessel to re-create the adipose tissue that typically surrounds the carotid artery [23].

Model plaques were designed to mimic 70 % stenosis in the 8 mm vessel. Plaques possessed an idealized anthropomorphic shape, resembling that of a small seed. Two different plaque models were designed. The first design modeled an atherosclerotic plaque that possessed an atheroma cap, IPH site, and a distinct lipid-rich necrotic core (into which hemorrhage had not yet infiltrated). The second design modeled a plaque with a much larger IPH site that had spread throughout the plaque’s lipid core. The atheroma cap was composed of 97.95 wt % deionized water, 2 wt % agar, and 0.05 wt % sodium azide. The lipid-rich necrotic core was mimicked using an oil-in-water emulsion (48.6 wt % canola oil, 48.6 wt % deionized water, 1.25 wt % lecithin, and 0.05 wt % sodium azide) solidified with 1.5 wt % agarose. All IPH sites were created using the same methemoglobin-mimicking material used in the size standard phantom. Plaques were fabricated using a series of 3D printed molds that were designed using computer-aided design software. To produce the multi-component plaques, a layer-by-layer casting approach was used (see Supplementary Fig. 1). Three identical replicates of each plaque design were made. Once a given plaque was complete, it was slid into the agar vessel to produce the final atherosclerosis phantom.

Before imaging, the lumen of each vessel was filled with perfluoro-n-hexane so that it would appear hypointense and thus match the appearance of nulled blood in MPRAGE images. Phantoms were then imaged using the MPRAGE sequences described below and the volume of IPH in each plaque replicate was measured.

### MR Imaging of Phantoms

Images were acquired using a 3 T Siemens Prisma scanner (Siemens, Erlangen, Germany), using a T1-weighted, 3D-MPRAGE carotid imaging sequence (adjusted to include a water-excitation pulse and blood nulling). Sequence parameters were TR = 1200 ms, TE = 4.64 ms, TI = 606 ms, flip angle = 10 °, NEX = 1, FOV = 228 × 270 mm, acquisition matrix size = 216 × 256, and slice thickness = 0.5 mm, 112 slices. To better visualize plaque features and accurately assess IPH size, a high-resolution MPRAGE sequence with an acquisition matrix size of 256 × 288, a FOV of 178 × 200 mm, a slice thickness of 0.6 mm, and NEX = 2 was also used.

T1 maps were acquired using the Siemens MapIt protocol (Siemens, Erlangen, Germany), which acquired images using a T1-weighted volumetric interpolated breath-hold examination (VIBE) sequence with two different flip angles (2 °, 10 °), TE = 1.71 ms, TR = 4.95 ms, acquisition matrix size = 192 × 154, FOV = 270 × 270, slice thickness = 2.0 mm, and 48 slices. Prior to T1 mapping, a B1 map was acquired to improve T1 map homogeneity, and T1 maps were calculated inline.

### MR Image Analysis

Mean T1 relaxation times for Gd^3+^ diffusion experiment hydrogels and the IPH size standard phantoms were calculated from corresponding stacks of T1 maps. To ensure partial volume effects and Gibbs artifacts did not affect T1 measurements, IPH sites smaller than 6 mm were not included when calculating means for a given hydrogel formulation. All T1 relaxation times are expressed as the mean ± standard deviation.

The mean MPRAGE signal intensities of IPH sites were calculated from the stack of high-resolution MPRAGE images. The corresponding uncertainty was taken to be ± standard error of the mean. These means were used to calculate the percent change in signal intensity for each of the nine IPH sites in the size standard phantom over the 31-day storage period.

Radial profiles of signal contrast for the cylindrical methemoglobin-mimicking hydrogels (from the Gd^3+^ diffusion experiments) were created by bisecting a cross-sectional MPRAGE image of the gel, calculating the contrast between each pixel along this line and the background agar, and then plotting the resulting contrast values as a function of radial position. Plots were acquired for a series of slices distributed throughout the phantom and the mean contrast values at each radial position calculated and graphed.

The mean cross-sectional area of each cylindrical site in the IPH size standard phantom was measured using the high-resolution MPRAGE images. The outer limit of each IPH site was taken as the location where the signal intensity dropped below the midpoint between the mean background intensity and the peak signal intensity of the IPH site (signal intensity of the inner 20% of the site). Anatomical IPH site volumes were measured from the high-resolution MPRAGE images. Each 2D plaque image in the relevant stack was automatically segmented for the IPH site (using the same threshold definition described above) and the sum of the selected voxels calculated. The calculated cross-sectional areas and anatomical IPH site volumes are reported along with the areas/volumes measured when the threshold signal intensity is set to the original threshold plus/minus standard deviation.

## Results

**Fig. 1.**
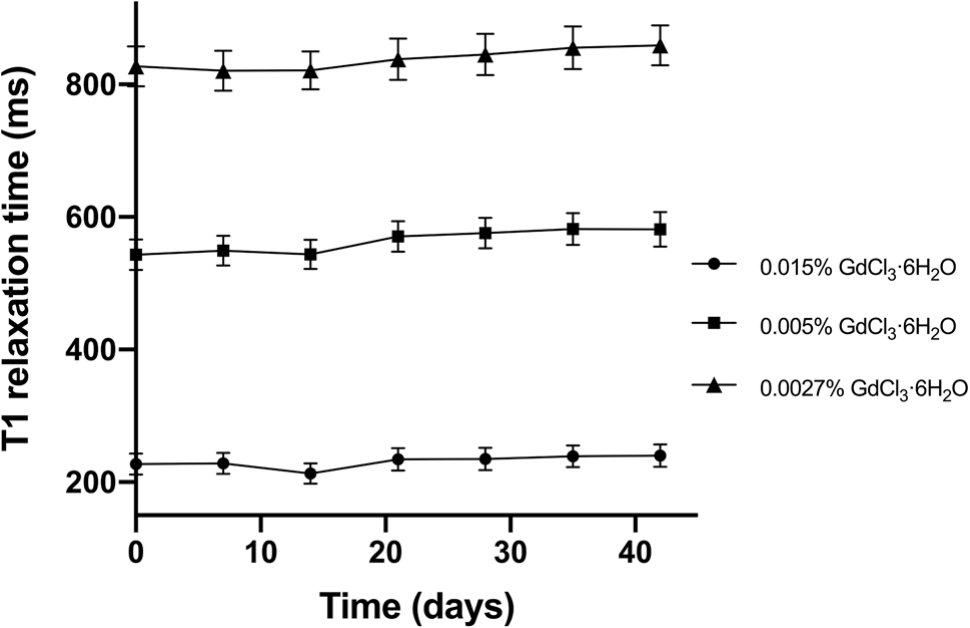
Mean T1 relaxation times for methemoglobin-mimicking hydrogels (containing sodium alginate) over the 42-day assessment period. Error bars indicate standard deviation.

**Fig. 2.**
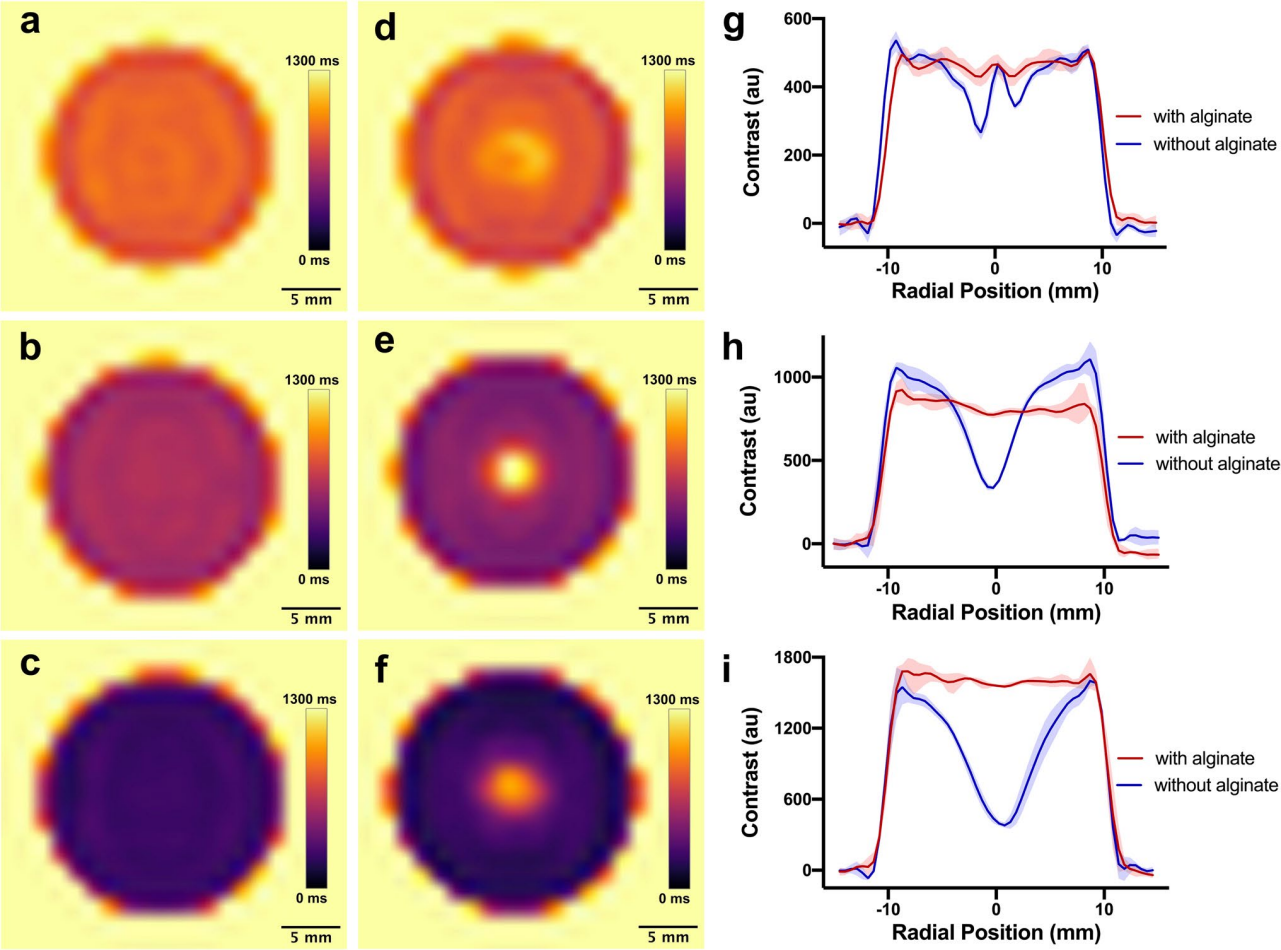
T1 maps of methemoglobin-mimicking hydrogels (20.7 mm diameter) with sodium alginate (**a–c**) and without (**d–f**), after an assessment period of 42 days. The circular sites shown in each image are surrounded by agar gel. MPRAGE contrast (**g–i**) (between the methemoglobin hydrogel and background agar) as a function of radial position for the methemoglobin hydrogels shown in (**a–f**). Shaded regions represent the maximum and minimum contrast values observed at a given radial position. GdCl_3_**·**6H_2_O concentrations in the methemoglobin gels were 0.0027 wt % for the top row (**a**, **d**, **g**), 0.005 wt % for the middle row (**b**, **e**, **h**), and 0.015 wt % for the bottom row (**c**, **f**, **i**).

Over 42 days, the mean T1 time of alginate-containing methemoglobin hydrogels remained near-constant (Fig. 1). The percent change in mean T1 time was independent of gadolinium concentration, measured as 5 ± 10 %, 7 ± 6 %, and 4 ± 5 % for the 0.015 wt %, 0.005 wt %, and 0.0027 wt % gadolinium(III) chloride hexahydrate hydrogels, respectively. Furthermore, T1 mapping showed that the alginate-containing gels remained spatially homogenous over time (42 days) (Fig. 2a–c). Conversely, doped hydrogels that did not include sodium alginate demonstrated a lengthening of T1 relaxation times near their center (Fig. 2d–f). The effect of sodium alginate on T1 relaxation properties was also evident when MPRAGE images of the gels were acquired (Supplementary Fig. 2) and signal intensity graphed as a function of radial position (Fig. 2g–i).

**Fig. 3.**
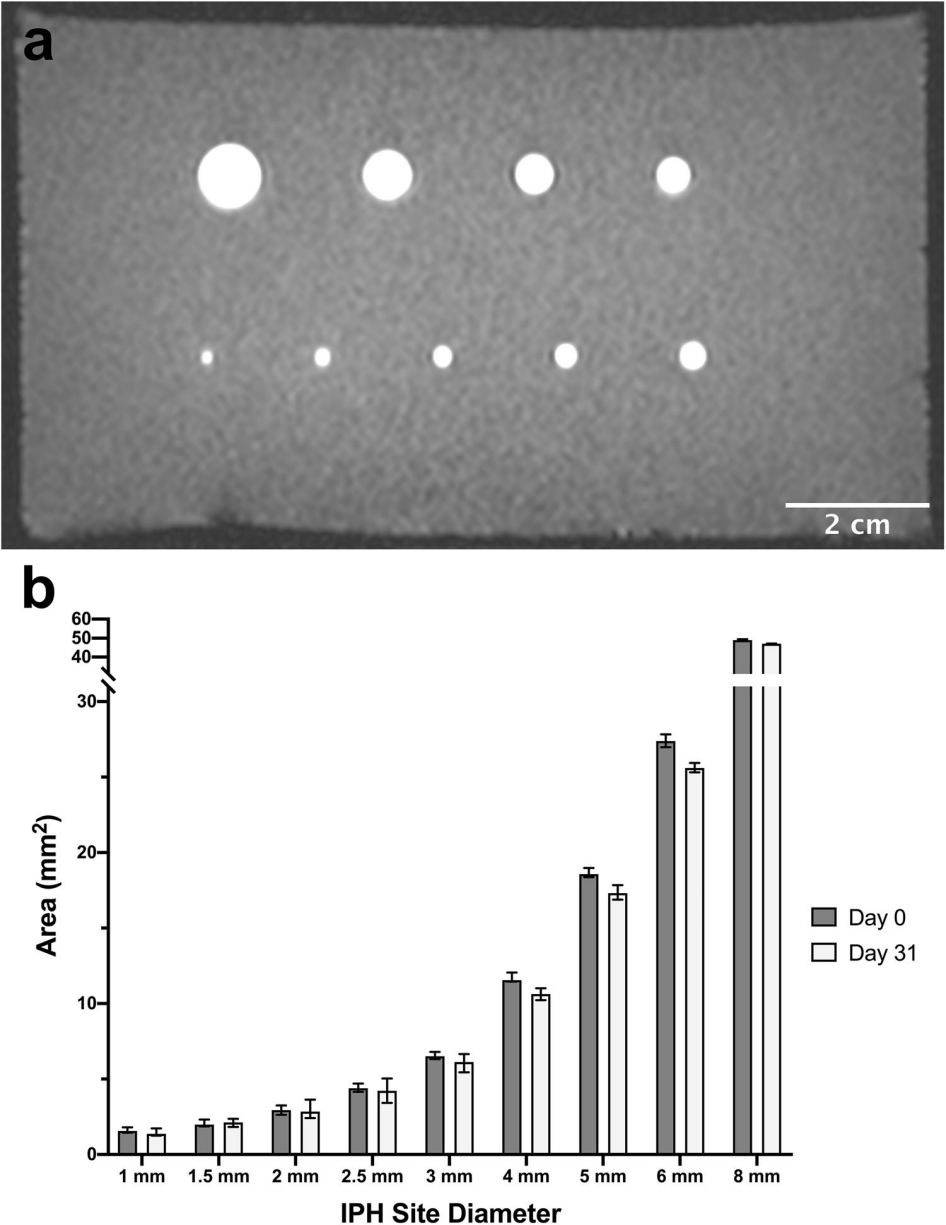
Characterization of the IPH size standard phantom: **a** Representative image of the phantom scanned using the high-resolution MPRAGE sequence. **b** Mean cross-sectional areas of the IPH sites, before and after a 31-day storage period.

The IPH size-standard phantom (shown in Fig. 3a) possessed 9 hyperintense mock-IPH sites, each with a circular cross-section. The methemoglobin-mimicking material used in the sites (with a GdCl_3_**·**6H_2_O concentration of 0.01 wt %) had an average T1 relaxation time of 335 ± 51 ms. Minimal changes were observed in the mean cross-sectional area of each IPH site over the 31-day assessment period (Fig. 3b). Across all sites, the average absolute change in cross-sectional area was 6 ± 3 %. The MPRAGE signal intensity of the sites also changed minimally over this time period. For IPH sites larger than 1.5 mm, the absolute change in signal intensity was no greater than ~ 6 % (Table 1). Smaller sites exhibited larger changes in signal intensity (decreases in signal intensity of 15 ± 2 % and 23 ± 2 % were recorded for the 1.5 mm and 1.0 mm IPH sites, respectively).

**Table 1:** Percent change in signal intensity of the IPH size standard phantom over the 31-day assessment period

IPH site diameter	Signal intensity change
8 mm	4.0 ± 0.9 %
6 mm	4 ± 1 %
5 mm	4 ± 2 %
4 mm	3 ± 2 %
3 mm	− 4 ± 3 %
2.5 mm	− 4 ± 2 %
2 mm	− 6 ± 2 %
1.5 mm	− 15 ± 2 %
1 mm	− 23 ± 2 %

**Fig. 4.**
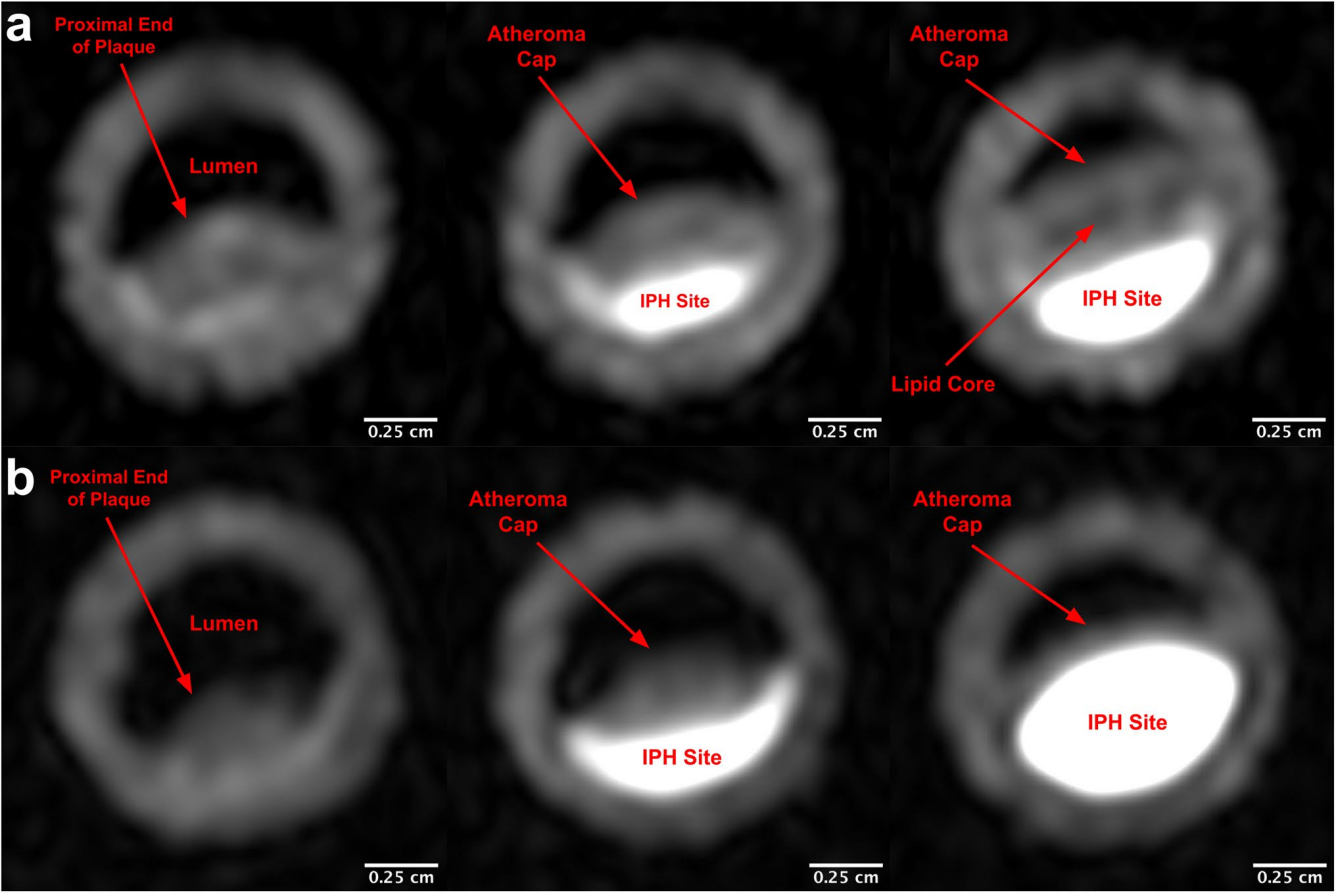
High-resolution MPRAGE images of the moderate IPH (**a**) and severe IPH (**b**) anatomical atherosclerosis models. The presented slices show plaque cross sections at different axial positions along the length of the mock vessel.

Representative slices of the anatomical atherosclerosis phantoms, modeling either moderate or severe IPH, are shown in Fig. 4 (for a set of slices that span the entire plaque, see Supplementary Fig. 3). The phantoms possessed distinct IPH sites; atheroma caps; and, in the case of the moderate IPH model, an identifiable lipid-rich necrotic core. The measured IPH site volumes for the 3 replicates of each phantom model are shown in Supplementary Fig. 4. This figure shows that for 3 independently synthesized phantoms of each type, IPH site volumes were nearly identical.

## Discussion

In this research, a time-stable material that mimicked the T1 relaxation properties of methemoglobin-laden IPH was developed and then used to fabricate two realistic IPH imaging phantoms; an IPH size standard phantom and an anatomical model of an IPH-positive plaque.

Results demonstrated that a methemoglobin-mimicking material that maintains near-constant T1 relaxation properties and homogeneity over a 42-day period can be synthesized by incorporating a minute amount (0.15 wt %) of sodium alginate into GdCl_3_-doped agarose hydrogels. This imaging stability indicates that the diffusion/migration of Gd^3+^ from the methemoglobin-mimicking material was limited, likely due to ionic bonding between Gd^3+^ ions and the anionic groups present on the alginate polymer. Notably, the material exhibited this stability when in direct contact with other hydrogels, without requiring encasement in an impermeable (*e.g.,* plastic or glass) barrier. Without the need for an impermeable barrier between the methemoglobin-mimicking material and other regions of the phantom, more realistic appearing phantoms can be created. Furthermore, the methemoglobin-mimicking material possesses the inherent castability of agarose-based hydrogels, thus allowing for it to be formed into a range of complex, end user–customizable shapes. It should also be noted that since this material was stable over a range of gadolinium concentrations, the gadolinium concentration can be easily tuned to match the T1 time of a variety of anatomical structures to fabricate other anatomical MRI phantoms.

In the IPH size standard phantom, mock-IPH sites appeared hyperintense on MPRAGE images, consistent with the appearance of methemoglobin deposits, over the full range of IPH site sizes (from 1 to 8 mm) [7]. The T1 time of the mock IPH sites also agreed well with measured values of clinical IPH [24]. Ideally, the mock-IPH sites would be perfectly time-invariant; however, this level of stability would require encasement using a glass or plastic barrier that would compromise phantom realism and ease of manufacture. Here, minimal change was detected in the size and signal intensity of the IPH sites over the 31-day assessment period, except in the most challenging case, where in smaller diameter sites (less than 2 mm), decreases in IPH signal intensity of 15 % or greater were observed. This is expected (and difficult to prevent) given their higher surface area to volume ratios facilitate more rapid Gd^3+^ diffusion compared to larger diameter sites. In addition, the mean cross-sectional area measured for the 1 mm IPH site (Fig. 3) was larger than the theoretical area of a circle with 1 mm diameter. This was likely due to the partial volume effect that reduced the peak signal intensity of the site, resulting in the calculation of a lower boundary threshold (see “MR Image Analysis” above) and increasing the measured area. Despite this, the change in the site’s area over the 31-day assessment period was small, thus permitting it to still serve as a static reference during scanner calibration. Based on its stability, the phantom is suitable for transport to and/or between multiple institutions for scanner calibration in a multicenter IPH imaging study. In future work, the phantom stability over longer time periods and the impact of different storage conditions will be assessed.

A simplified cylindrical shape was selected as the geometry of the anatomical vessel phantom even though atherosclerotic plaques are often concentrated around the carotid bifurcation [25]. Furthermore, the idealized anthropomorphic shape chosen for the plaques was not identical to what would be observed pathologically [26]. While selecting a simplified geometry allowed for facile fabrication, it also resulted in a less anatomically realistic carotid replica. Despite these limitations, the fabricated anatomical atherosclerosis phantoms closely resembled clinical plaques when imaged with the MPRAGE sequence. In addition to a hyperintense IPH site, the lumen and surrounding adipose tissue (modeled using paraffin wax) appeared hypointense, replicating the effects of the blood nulling and water excitation techniques employed in carotid scans [7, 27]. The lipid-rich necrotic core (only identifiable in the moderate IPH model) appeared just slightly darker than the vessel wall, consistent with its appearance clinically using fat suppressed, T1-weighted images [28]. Although the atheroma caps of the plaque replicas were made from the same material as the vessel wall (a 2 wt% agar hydrogel), in pathological plaques, the atheroma cap is distinct in composition, being primarily composed of fibrous connective tissue. Nonetheless, this distinction is not reflected in the phantom as a distinct fibrous cap is typically difficult to identify on T1-weighted MPRAGE images [29]. In addition to realism, a final important feature of the phantoms is that the fabrication process has a high fidelity and reproducibility. The measured IPH site volumes for each of the three replicates of the two phantom models produced were nearly identical.

By combining this phantom, which accurately models the different components of a plaque, along with one that can be used to better standardize IPH size measurements, one possesses a novel set of tools which will allow for scanner calibration in IPH imaging studies. These phantoms would be used to ensure each scanner in a multicenter study produces standardized images through a calibration process wherein the phantoms are imaged on a given scanner, key metrics of the resulting images (such as IPH site volume/area and signal intensity) are measured via image analysis, and then scanning parameters are adjusted until these metrics and the overall appearance of the phantoms are consistent with reference images. Given its demonstrated stability over time, the size standard phantom would be well-suited for quantitative calibration of IPH site signal intensity and area, while the anatomical atherosclerosis phantom could be used to standardize overall image appearance and 3D IPH volume measurements. In future work, a step-by-step scanner calibration protocol that utilizes these phantoms will be developed and tested. This future study will involve imaging the phantoms on various scanning platforms and at multiple institutions; assessing the differences in overall appearance, signal intensity, and IPH site size measured across scanners; and then attempting to use the phantoms to calibrate the scanners so standardized images are produced.

## Conclusions

A stable, castable material that accurately represents methemoglobin in MRI was developed. It was used to fabricate a novel phantom that modeled IPH sites of varying diameters (1–8 mm) which were found to maintain both their size and signal intensity over 31 days. Reproducible replicas of IPH-positive atherosclerosis were also created and shown to accurately model the appearance of common plaque components. In combination, these phantoms represent a powerful tool to standardize IPH imaging protocols and thus facilitate multicenter IPH imaging.

## Supplementary Information

Below is the link to the electronic supplementary material.

Supplementary file1 (DOCX 684 KB)
