# Supplementary material for: Fabrication of Customizable Intraplaque Hemorrhage Phantoms for Magnetic Resonance Imaging
Source: Mol Imaging Biol. 2022 Apr 29;24(5):732–9. doi: 10.1007/s11307-022-01722-4 (PMC9581813; doi:10.1007/s11307-022-01722-4)
Supplement: Supplementary file 1 — Supplementary file1 (DOCX 684 KB) [file 11307_2022_1722_MOESM1_ESM.docx]

**Fabrication of Customizable Intraplaque Hemorrhage Phantoms for Magnetic Resonance Imaging:**

**Electronic Supplementary Material**

Matteo A. Bomben,^1,5^ Alan R. Moody,^2,6^ James M. Drake^1,4,5^ and Naomi Matsuura^2,3,4^

^1^Department of Mechanical and Industrial Engineering

^2^Department of Medical Imaging

^3^Department of Materials Science and Engineering

^4^Institute of Biomedical Engineering, University of Toronto, Toronto, Ontario, Canada

^5^Centre for Image Guided Innovation and Therapeutic Intervention, The Hospital for Sick Children, Toronto, Ontario, Canada

^6^Sunnybrook Hospital, Toronto, Ontario, Canada


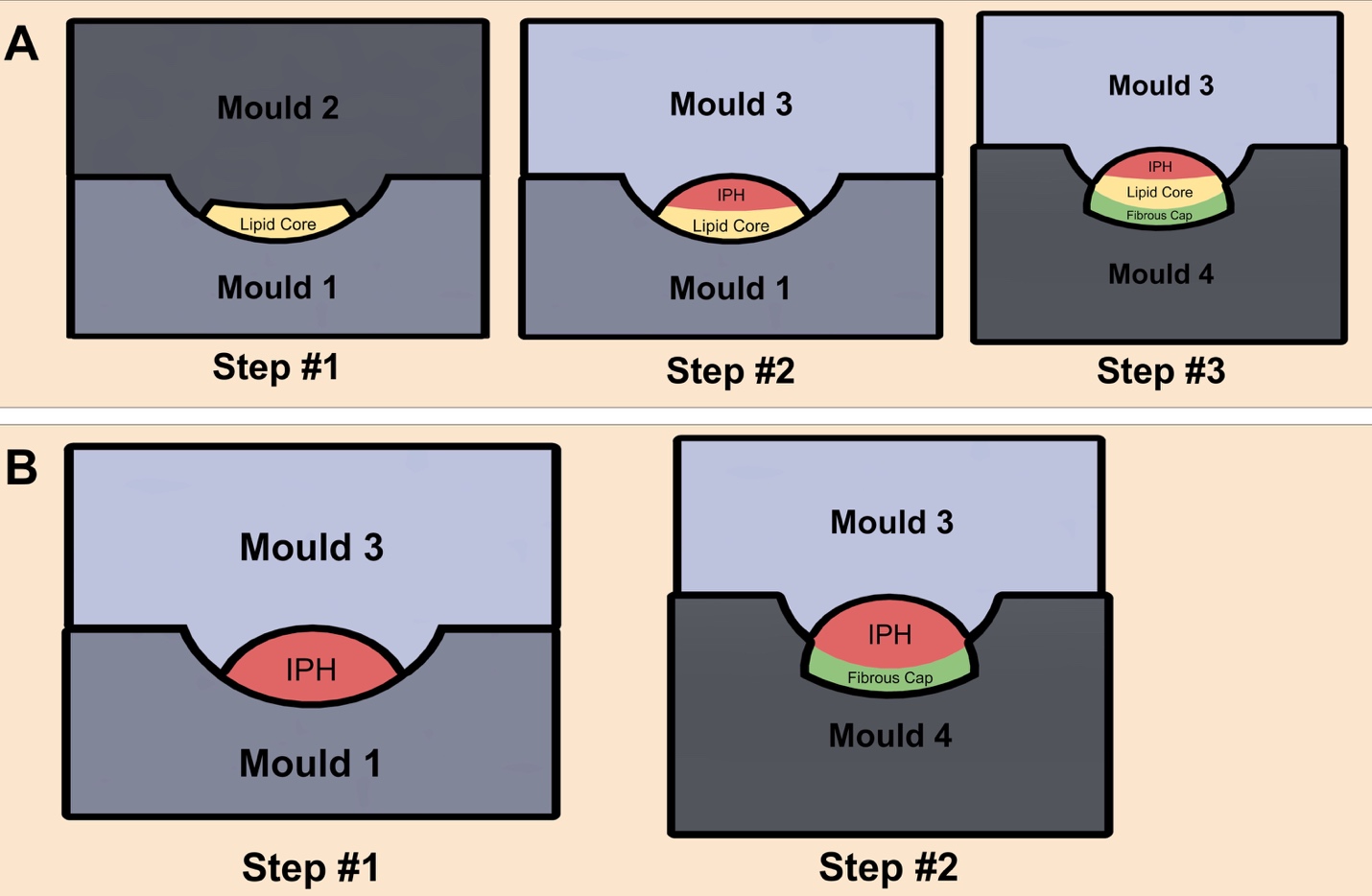


**Supplementary Fig. 1:** (a) The casting steps used to create a plaque that exhibits moderate IPH. Step #1: the lipid core is cast between Mould 1 and Mould 2. Step #2: Mould 2 is replaced with Mould 3 and the resulting void is filled with the methemoglobin-mimicking material to form the IPH site. Step #3: while still attached to Mould 3, the lipid core and IPH site are transferred to Mould 4 and the fibrous cap is added. (b) The casting steps used to create a plaque that exhibits severe IPH. To create the larger IPH site, the initial casting step shown in (a) is bypassed and the entire void between Mould 1 and Mould 3 is filled with the methemoglobin-mimicking material.


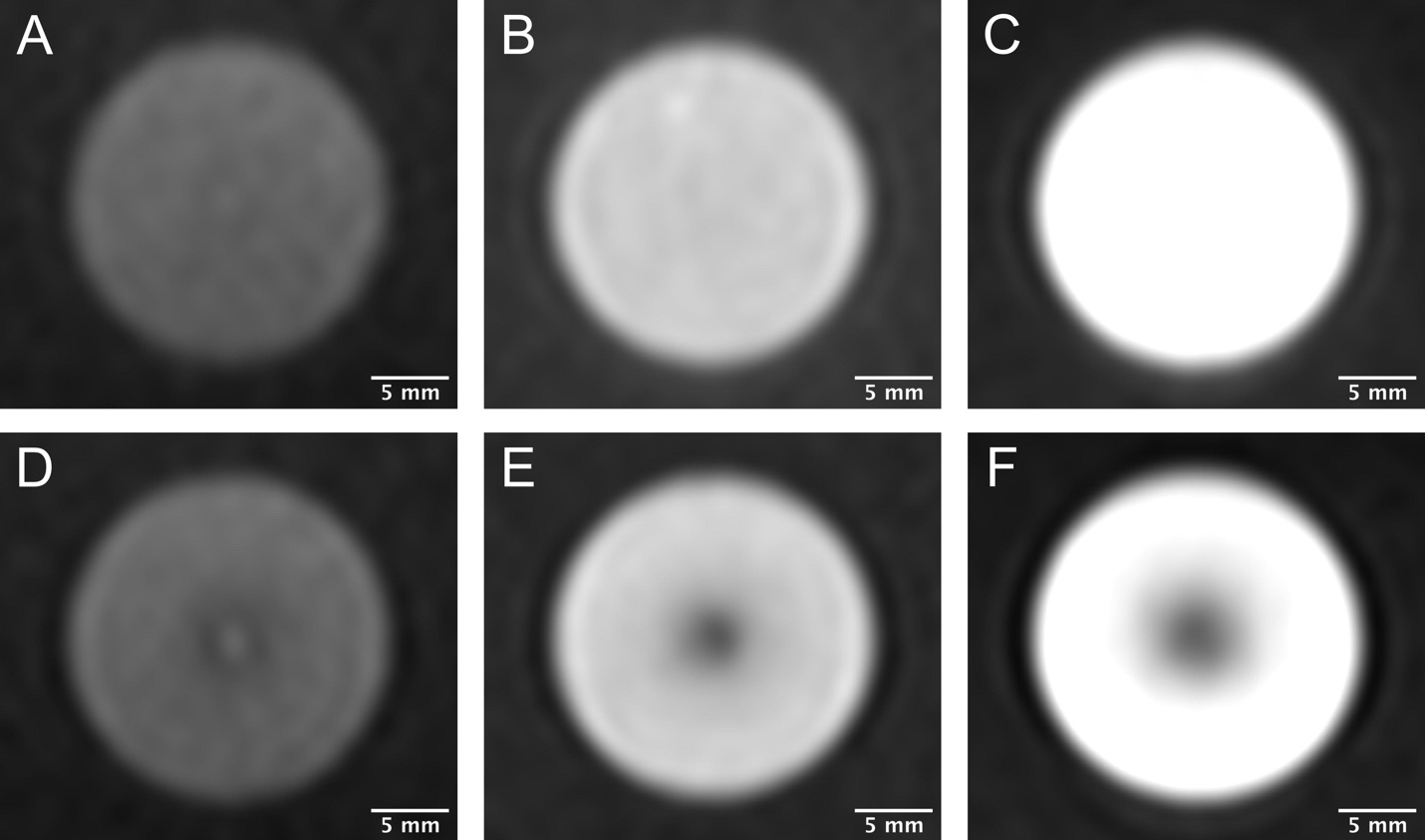


**Supplementary Fig. 2:** Cross-sectional MPRAGE images of the methemoglobin-mimicking hydrogels with sodium alginate (a-c) and without (d-f). GdCl_3_**·**6H_2_O concentrations in these gels were 0.0027 wt%, 0.005 wt%, and 0.015 wt% for a-c and d-f, respectively.


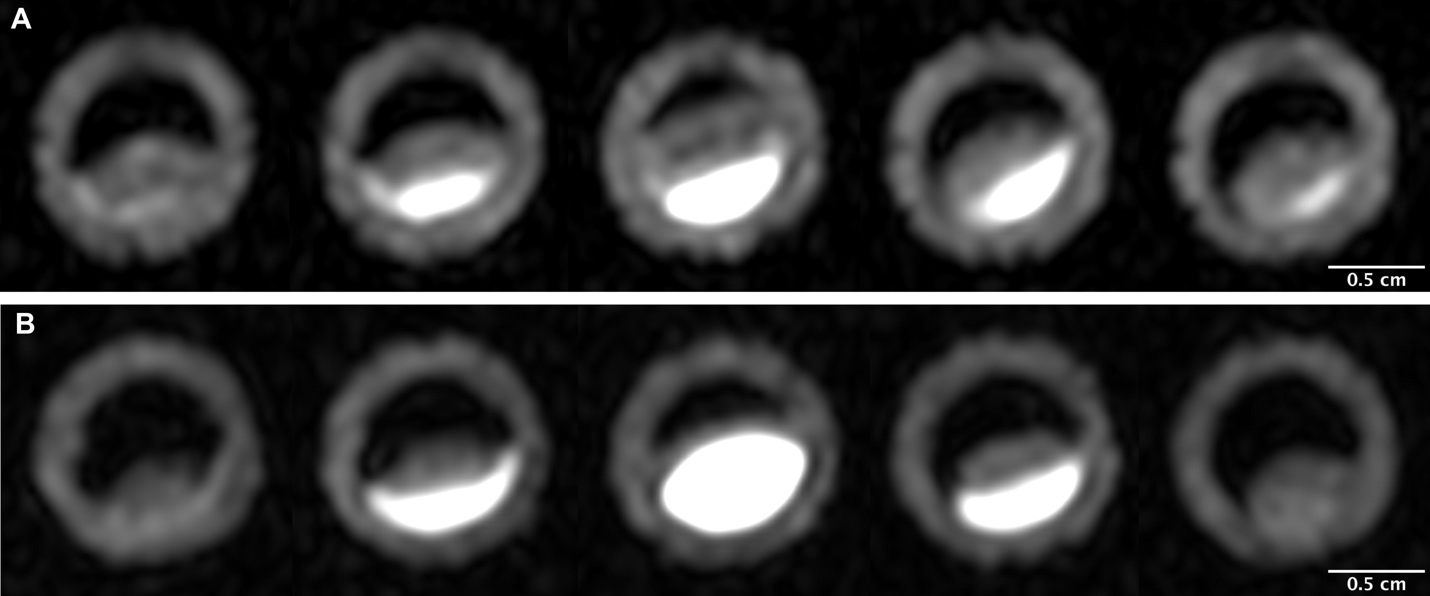


**Supplementary Fig. 3:** Sets of high resolution MPRAGE slices showing both anatomical atherosclerosis phantom models. The moderate IPH model (small IPH site) is shown in (a) and the severe IPH model (large IPH site) is shown in (b).


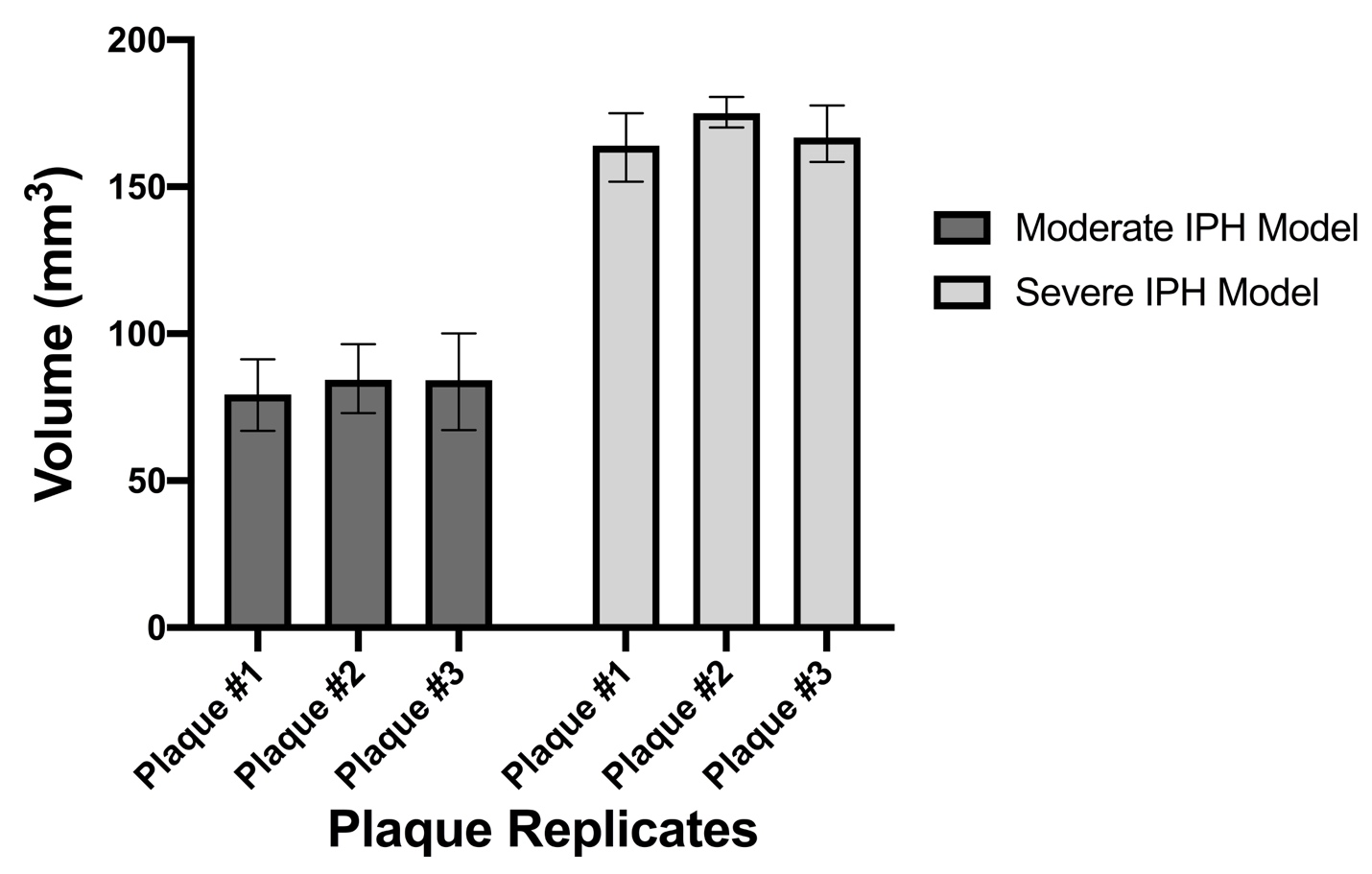


**Supplementary Fig. 4:** Measured IPH site volumes for each anatomical atherosclerosis phantom replicate produced.
